# Bilateral Compartment Syndrome in Intravenous Drug Abuse

**DOI:** 10.7759/cureus.3683

**Published:** 2018-12-04

**Authors:** Justin Loloi, Alex T Burton, Leonard T Walsh, Nitasa Sahu, Rohit Jain

**Affiliations:** 1 Internal Medicine, Penn State Health Milton S. Hershey Medical Center, Hershey, USA; 2 Orthopaedic Surgery, Penn State Health Milton S. Hershey Medical Center, Hershey, USA

**Keywords:** intravenous, drug, compartment, bilateral, reperfusion, immobilization, syndrome, opioid, palsy, pressure

## Abstract

Compartment syndrome is an orthopedic emergency in which the neurovasculature of the extremity is compromised. Typically, it presents unilaterally and is the consequence of major trauma to the extremity in the form of fracture. However, more uncommon etiologies of compartment syndrome have been reported, which includes reperfusion injury, burns, and congenital or acquired bleeding disorders.

We present an extremely rare case of bilateral posterior thigh compartment syndrome thought to be due to intravenous drug abuse (IVDA) causing prolonged ischemia with subsequent reperfusion. This case is particularly relevant in today’s clinical setting given the current opioid epidemic and subsequent rise in intravenous drug use.

## Introduction

Compartment syndrome is a condition in which the circulation and function of tissues within a closed space are compromised secondary to increased intra-compartment pressure. It typically presents acutely and unilaterally as a result of trauma and if not quickly managed may result in cellular anoxia, muscle ischemia, and tissue necrosis [1]. The most common etiologies include trauma resulting in fractures and contusions, bleeding disorders and burns [2]. Non-traumatic causes of compartment syndrome are far more uncommon. Compartment syndrome secondary to intravenous drug abuse is a phenomena that has only been reported in a handful of cases [3]. Given the worsening opioid abuse epidemic, complications of overdose including compartment syndrome can be expectedly on the rise.

This report illustrates, to our knowledge, the first-reported case of a patient presenting with bilateral posterior thigh compartment syndrome secondary to immobilization in the setting of intravenous drug abuse.

## Case presentation

A 41-year-old male with a past medical history of intravenous drug abuse (IVDA) presented to our emergency department as a transfer following two days of medical management of rhabdomyolysis at an outside community hospital. He initially presented with weakness and numbness of the lower extremities. He had been using intravenous heroin and was lying supine on a floor for approximately 24 hours.

Upon presentation to our emergency department, the patient had pain in his bilateral lower extremities and no appreciable motor function below the level of the knee. He was in mild distress with a temperature of 37°C, pulse of 104 beats per minute, blood pressure of 146/56 mmHg, respiratory rate of 18 and oxygen saturation of 97% on room air. Examination of the lower extremities revealed erythema and tension in the bilateral posterior thigh compartments. In contrast, the medial thigh compartments bilaterally along with all four leg compartments were soft. Gluteal and buttock compartments were soft and compressible bilaterally. Motor strength testing revealed 1/5 strength bilaterally with manual testing of ankle dorsiflexion and plantarflexion, and knee flexion and extension. The patient was found to have bilateral sciatic nerve palsies, with no sensation bilaterally in the tibial nerve, deep and superficial peroneal nerve distributions. Both legs were warm and pink with brisk capillary refill along with 2+ dorsalis pedis pulses bilaterally. Provocative testing demonstrated pain with passive range of motion of the knees bilaterally and no pain with passive ankle dorsiflexion and plantarflexion. Lab work was significant for a creatinine phosphokinase (CPK) of 231,360 U/L.

Given the high suspicion for compartment syndrome, the patient underwent compartment pressure testing via Stryker pressure monitor system. Diastolic blood pressure recording measured 75 mmHg. The right posterior thigh compartment measured 75 mmHg and left posterior thigh compartment measured 30 mmHg. Based on the history and physical examination findings, the patient was diagnosed with bilateral thigh compartment syndrome likely secondary to his recent IVDA and subsequent immobilization and was taken to the operating room for urgent bilateral posterior thigh fasciotomies.

**Figure 1:**
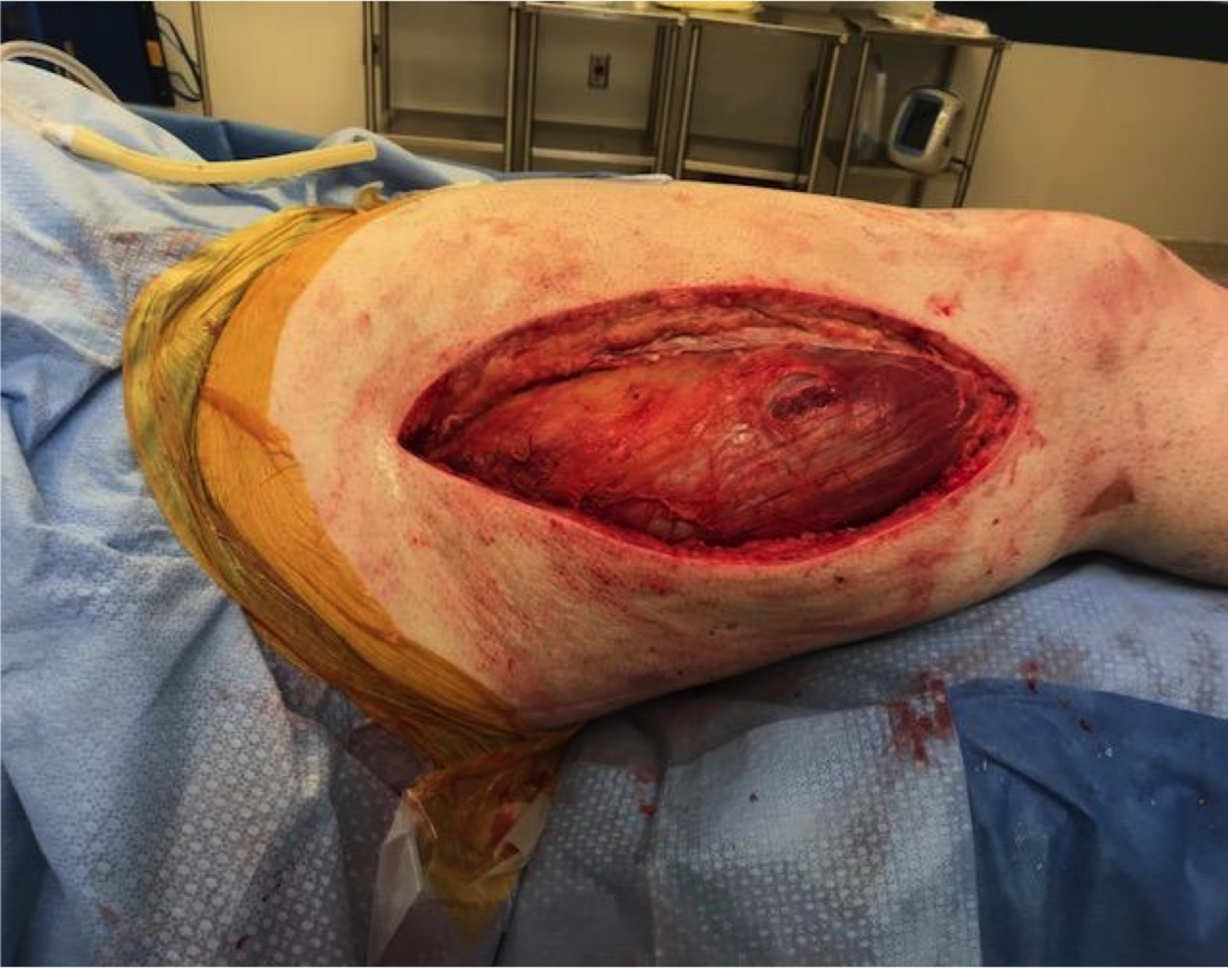
Release of the anterior and posterior compartments of the right thigh.

In the operating room, the right posterior compartment was approached first. The intermuscular septum was released with the fascia along the posterior compartment for full release of both the anterior and posterior compartments of the right thigh (Figure 1).

**Figure 2:**
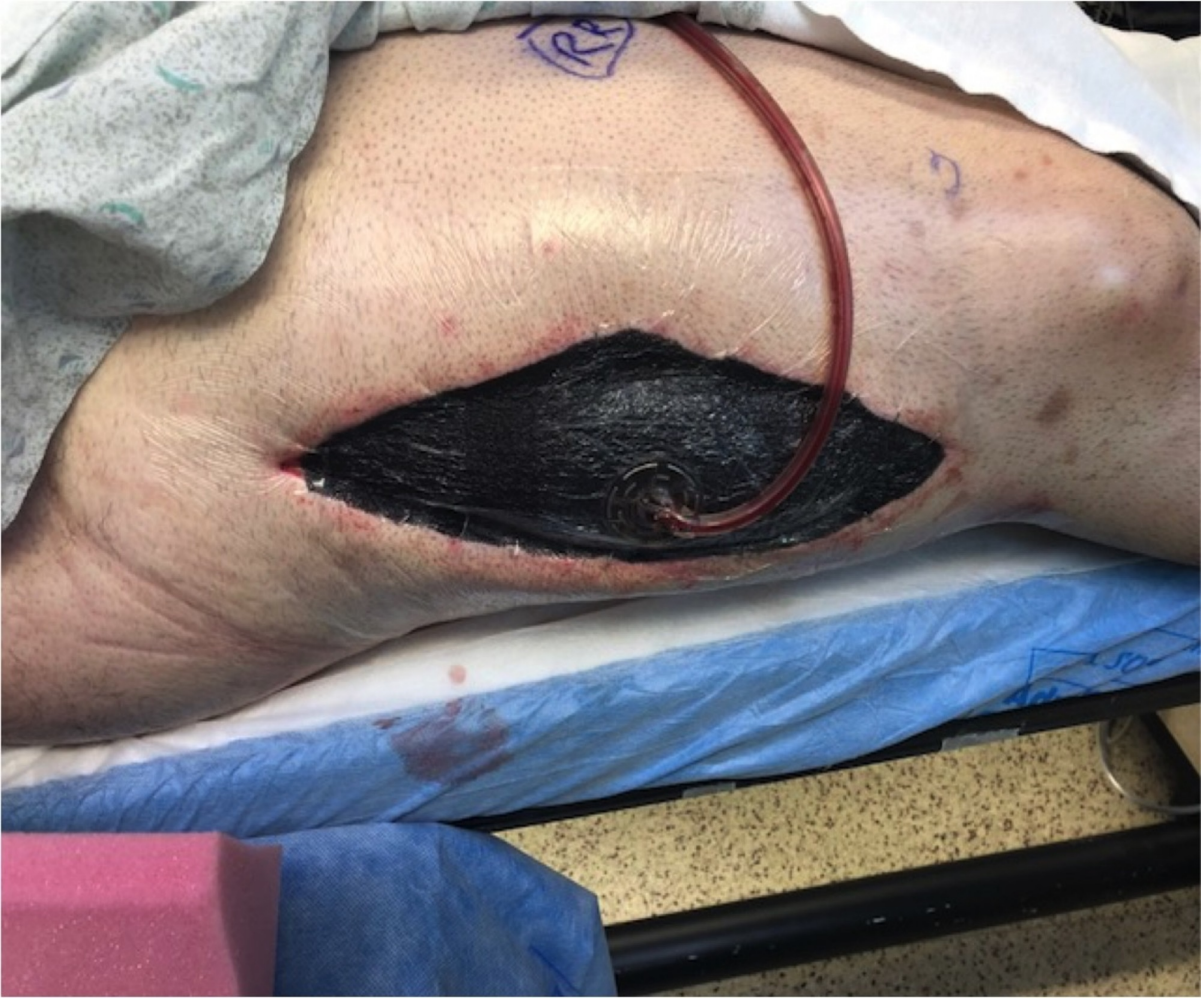
Negative pressure wound VAC placed over the compartment fasciotomy. VAC: Vacuum-assisted closure

Following fasciotomy, cautery was used to confirm the viability of the exposed muscles in the anterior and posterior compartments. The skin surrounding the incision for the compartment release was too tense for safe closure, and so it was decided to place a wound vac. A 22 x 8-cm negative pressure vacuum-assisted closure (VAC) dressing was then placed over the wound (Figure 2).

Attention was then paid to the left thigh. Compartment pressures were rechecked. The left posterior thigh compartment measured 30 mmHg. Given these findings, the decision was made to forego the compartment release due to the morbidity associated with fasciotomies.

Serial wound vac changes were then carried out on postoperative day (POD) two and four. On POD six, we removed the wound vac and placed a DermaClose device for assistance with skin closure. On POD 10, his skin edges were closable and so he underwent complex wound closure approximately 20 cm in length.

Throughout operative treatment and recovery, the patient was given antibiotics and developed no further complications. His CPK levels returned to normal. He was discharged on antibiotics and was given counseling on the potential adverse effects of IV-drug abuse. He had a favorable recovery at two-week follow-up.

## Discussion

Compartment syndrome is a debilitating orthopedic emergency that, if not promptly managed, can lead to long-term consequences such as limb amputation. It is often a clinical diagnosis and typically affects the unilateral limb prompting emergent fasciotomy to relieve pressure within the compartment. Following extremity ischemia as seen in traumas, burns, and even prolonged compression in a obtunded or comatose patient, reperfusion of the tissues may result in compartment syndrome [4]. This is because of inflammatory responses following reperfusion which may damage the muscle tissue and microvasculature [5]. Here, we present a novel case of bilateral posterior thigh compartment syndrome following a prolonged period of immobilization on the ground secondary to IVDA. Particularly in an era of increased use of opioids and IV drugs, this case highlights the importance of counseling patients on the risks and complications associated with IV drug use, which may represent a modifiable risk factor for the development of compartment syndrome.

Bilateral compartment syndrome of the posterior thigh is sparsely documented in the literature but has been documented in the setting of intense exercise and prolonged lithotomy position [6,7]. In both reports, the patients underwent emergent bilateral fasciotomy. In our case, because of improved intraoperative pressure readings on the left posterior thigh, the decision was made to forego the fasciotomy to minimize the comorbidities associated with the procedure. However, the data available suggests that bilateral compartment fasciotomy represents the mainstay of treatment in this type of unique patient presentation.

IV-drug abuse is a national crisis that is on the rise [8,9]. IV-drug abuse is associated with cutaneous complications such as cellulitis, abscess formation, ulcers, and necrotizing fasciitis and may result in systemic disease such as hepatitis C, human immunodeficiency virus (HIV), and infective endocarditis [10]. Drugs of abuse such as IV-heroin have been associated with causing prolonged immobilization, which can result in muscle compression and ischemia, and upon reperfusion of the tissues, may result in compartment syndrome [11,12]. Our patient presented with clear signs of acute compartment syndrome after IV-heroin injection, causing immobility for a prolonged period of time suggesting a reperfusion syndrome. The patient was likely unconscious or in an altered mental status during the cardinal symptoms of compartment syndrome, which we believe was bilateral given his bilateral sciatic nerve palsy. However, at the time of transfer and presentation to our institution, the patient’s compartment syndrome was acquiescing on the left side.

It is important to note that this case represents a late presentation compartment syndrome in that if he was seen on day one, we likely could have opened his posterior compartment and prevented a sciatic nerve palsy. However, he came on day three after initial presentation and by the time we saw him, he was only in mild pain and distress. Even in the setting of improvement in extremity pain upon presentation to our center, we opted to undergo compartment release on the right given the patient’s clinical exam and compartment pressure. He was ultimately clinically stable throughout recovery and at follow-up.

This phenomenon of IV-drug use causing a reperfusion-injury compartment syndrome has yet to be described in the literature. Therefore, it is important for clinicians to recognize IV-drug abuse as a possible risk factor in the development of compartment syndrome. It is important to note that appropriate patient counseling may reduce the risk of future incidents.

## Conclusions

We present, to the best of our knowledge, the first report of bilateral posterior thigh compartment syndrome secondary to prolonged immobilization following IV-drug abuse. Given the steady rise in intravenous drug use, we recommend drug abuse be sought in the differential causes of acute compartment syndrome. With prompt recognition of this key social factor as a cause of this debilitating condition, we may provide our patients with counseling to decrease the risk of future occurrences.
